# Keystone Veterinary Medical Association

**Published:** 1895-11

**Authors:** W. L. Rhoads

**Affiliations:** Secretary


Secretary.

---

### KEYSTONE VETERINARY MEDICAL ASSOCIATION.

THE October meeting was called to order by President Lintz at the new and more commodious quarters on the Northwest corner of Broad and Filbert Streets, at 8.30, Tuesday the 8th inst. This move was imperative, as the increase in attendance of both members and visitors had outgrown the pleasant environments of Dr. Hoskins's office. The members present were Drs. F. Bridge, Charles T. Goentner, W. H. Hoskins, J. R. Hart, W.

S. Kooker, Charles Lintz, W. L. Rhoads, L. Pearson, and H. P. Eves. Dr. Allen was also present.

Dr. Hoskins, as chairman of the Legislative Committee, reported having received several communications from veterinarians now located in other States who wish to locate here, and would like to know what laws they must comply with.

Dr. Hart, as chairman of the Auditing Committee, reported the accounts satisfactory and the treasury in a condition to declare a dividend.

The revision of the Constitution and By-laws, that they might better meet the present and future needs of the Association, was now considered, and after some discussion they were adopted as revised.

Dr. Hoskins read a very interesting paper entitled "What I Have Found in Veterinary Associations." He thus brought out many pungent truths, which more clearly portrayed the inestimable worth of the Association as a factor for the protection and advancement of veterinary science, as well as the personal advantages to the individual member.

Dr. Eves reported a case of excessively contracted flexor tendons in colt one month old. This elicited considerable discussion and much valuable information.

Dr. Allen cited a case of farcy which had been tested with mallein and promised to give a detailed account at the next meeting. This brought into discussion the reliability of mallein as a diagnostic agent. It was maintained by a majority present, on account of variations in its manufacture, it was not as a rule thoroughly reliable, while tuberculin was held in higher repute than ever before, Prof. Nocard claiming a universal accuracy, while the Massachusetts commission claim less than one per cent. of mistakes with its use.

This being the annual meeting, the nomination and election of officers was in order. There being but one nominee for each office, the Secretary was directed to cast the ballot, and the election was declared unanimous, as follows: President, John R. Hart; Vice-President, W. S. Kooker; Treasurer, Francis Bridge; Secretary, W. L. Rhoads; Directors, Hiram P. Eves, Charles T. Goentner, Charles Lintz, Leonard Pearson, W. Horace Hoskins. After the new officers had assumed their duties the meeting adjourned to reconvene at the same place, Tuesday evening, November 12, 1895.

W. L. RHOADS,
Secretary.

---

